# Bat Astroviruses: Towards Understanding the Transmission Dynamics of a Neglected Virus Family

**DOI:** 10.3390/v9020034

**Published:** 2017-02-21

**Authors:** Kerstin Fischer, Vinícius Pinho dos Reis, Anne Balkema-Buschmann

**Affiliations:** Friedrich Loeffler Institut, Institute of Novel and Emerging Infectious Diseases, Suedufer 10, 17493 Greifswald-Insel Riems, Germany; Vinicius.reis@fli.de

**Keywords:** astroviruses, bats, zoonotic potential

## Abstract

Bats belong to the order Chiroptera that represents the second largest order of mammals with more than 1200 species and an almost global distribution. Environmental changes and deforestation have severely influenced many ecosystems, intensifying the contact between wildlife and humans. In recent years, bats have been found to harbor a number of different viruses with zoonotic potential, as well as a great diversity of astroviruses, for which the question of zoonotic potential remains unanswered to date. Human astroviruses have been identified as the causative agent for diarrhea in children and immunocompromised patients. For a long time, astroviruses have been considered to be strictly species-specific. However, a great genetic diversity has recently been discovered among animal and human astroviruses that might indicate the potential of these viruses to cross species barriers. Furthermore, our knowledge about the tissue tropism of astroviruses has been expanded to some neurotropic strains that have recently been shown to be responsible for encephalitis in humans and livestock. This review gives an overview on what is known about astroviruses in bats, humans and livestock, especially bovines and pigs. Future research activities are suggested to unravel astrovirus infection dynamics in bat populations to further assess the zoonotic potential of these viruses.

## 1. Introduction and Background

Astroviruses were first discovered in stool samples of infants suffering from diarrhea in 1975 [1]. Since then, our knowledge about the molecular and phenotypic characteristics of these viruses, on the viral pathogenesis and on the spectrum of susceptible hosts has been considerably expanded. Astroviruses belong to the *Astroviridae* family which is, according to the International Committee on Taxonomy of Viruses (ICTV), divided into two genera: *Mamastrovirus*, including 19 species, designated *Mamastrovirus 1–19*; and *Avastrovirus* including three species, formerly assigned as turkey, chicken and duck astrovirus. This virus family comprises a diverse group of small, non-enveloped single-stranded RNA viruses of positive polarity with a characteristic star-like appearance [2,3,4]. The genome consists of 6.17 to 7.72 kb with a 5′untranslated region (UTR) that is followed by three open reading frames (ORFs), a 3′UTR and eventually a poly-A tail [5,6]. ORF1a and ORF1b encode non-structural polyproteins 1a and 1b that include a protease and the conserved RNA-dependent RNA polymerase (RdRp) whereas ORF 2 encodes a more divergent structural capsid protein [3,7]. Altogether 19 species of mamastroviruses, have been identified with a wide geographic distribution in a great number of domestic animals, in wildlife including bats, as well as in humans [3,5,8,9,10,11,12,13,14,15,16]. Most of the infections caused by astroviruses are assumed to be asymptomatic but, depending on the affected species, the age and immunological status of the affected host, an infection can also be associated with diarrhea, hepatitis, nephritis or, more recently, even encephalitis [17,18]. In bats, astroviruses were mostly found in apparently healthy animals. Since 2008, a growing number of bat species have been found to carry astroviruses with a noticeable prevalence and diversity [11,19,20,21,22,23].

Bats are frequently considered the reservoir host for a broad variety of newly emerging viruses, especially in the tropics, although their general role in the epidemiology and spillover of zoonotic viral diseases is still not fully understood [24,25]. Some of these newly emerged viruses such as corona-, henipa- and filoviruses are zoonotic and show high pathogenic potential in humans [25,26,27,28,29]. As generally assumed for the reservoir hosts, bats do not develop severe clinical symptoms upon these viral infections. The reasons are still not fully understood and little is known about the immune system of bats and its interaction with pathogens. As the only flying mammals, bats have evolved special anatomical and physiological characteristics. Several of them appear to be relevant for their role as reservoir hosts of viral agents. As opposed to the reduced body temperature when resting, the body temperature of bats may increase during flight to above 40 °C, which is thought to mimic a fever [30]. On the other hand, the reduced body temperature and low metabolic rate during hibernation or torpor have been discussed to negatively affect the efficient immune response to infections. This may impair viral clearance, and therefore, by transmission to juvenile bats born after hibernation, may even cause virus persistence in the affected colony [31,32,33]. The roosting of certain bat species in gatherings of thousands if not millions of individuals is thought to facilitate high intra- and interspecies contact rates that might allow efficient virus transmission [31,34]. Deforestation, growing urbanization and environmental changes have not only destroyed great parts of the bats’ habitats, but have also increased their interactions with humans and livestock [35,36,37]. To analyze the potential health risk for humans, it has become important to study the ecology and the zoonotic potential of viruses found in bats. This review gives an overview on what is known about astroviruses in bats and their potential to cross species barriers to humans and/or livestock.

## 2. Astroviruses Detected in Bats across the Globe

**Figure 1:**
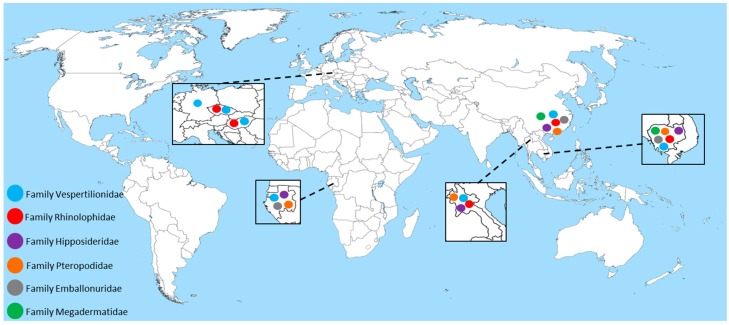
Geographic distribution of study sites for the detection of astrovirus-related RNA in bats. All of the sampled bat families are depicted in colored dots in the respective countries where information on species, sample size and region was available. Source of map: [41].

Within the last decade, many studies have been performed to investigate the occurrence and genetic diversity of astroviruses in several bat species from different regions around the world [11,15,19,20,21,22,23,38,39,40]. Although no data regarding the occurrence of astroviruses in bats are available for large geographical areas worldwide, the relatively few published studies were able to reveal astrovirus sequences for one-third of the known bat families (Figure 1). The primary study addressed a PCR-based screening of rectal swabs collected from apparently healthy insectivorous bats belonging to nine different species in Hong Kong [11]. Chu et al. developed a semi-nested reverse transcription PCR (RT-PCR) assay and observed overall high detection rates of 54% in fresh fecal samples at different times of the year and a notable genetic diversity of the detected astrovirus sequences [11]. The semi-nested RT-PCR assay laid the groundwork for many subsequent studies on the occurrence of astroviruses in bats. All these studies share their focus on phylogenetic analyses based on RT-PCR-derived partial genome sequences, while virus isolates and/or full-length genomes are still lacking (Table 1) [15,19,20,21,22,23,38,39,40]. Chu et al. successfully sequenced up to three-quarters of one bat astrovirus genome from a rectal swab sample in their study [11]. The genome (AFCD337) has been published in public databases and has served as a prototype for first phylogenetic analyses and classification [11]. Since then, a variety of partial sequences of shorter length have been published from numerous studies, displaying varying degrees of (amino acid) sequence similarities.

Samples tested positive for the presence of astrovirus genomes mainly included fecal samples but also urine and saliva/oro-pharyngeal swabs [11,15,19,20,21,22,23,38,39] albeit cross-contamination of fecal and urine samples, for instance, cannot be completely ruled out. In the case of positive saliva samples, fecal samples from the same animals were mostly positive as well [11]. Some of the analyzed samples were tissue samples taken from carcasses [15]. Fecal samples were collected from apparently healthy bats, and partially resulted in significant detection rates of astroviruses in certain bat species [11,19,20,21,22,38]. These findings raised the question whether astroviruses might persistently infect bats without causing any clinical symptoms, which has also been observed for many highly pathogenic viruses such as henipaviruses and filoviruses. Due to the lack of virus isolates and experimental data, the pathogenicity as well as the shedding pattern of astroviruses in bats is not yet understood. However, the information that can be drawn from field data will always be limited since a daily sampling scheme to fully unravel shedding patterns is incompatible with the welfare of the animal. Multiple samplings of tagged bat individuals over consecutive years in Germany revealed that most of the animals that were initially tested positive in RT-PCR were found not to be positive again after a period of between three and 36 months [19]. If bat individuals tested positive again, the sequences varied significantly, suggesting a successful clearance of the first astrovirus strain followed by an infection with a second strain which has not yet been recognized by the immune system. In another study of a *Myotis myotis* colony in Germany over three consecutive years, Drexler et al. observed one unique peak of astrovirus shedding per year with the exception of a second peak associated with parturition in the third year [38]. Notably, the authors assumed that each year, the first peak resulted from the introduction of a certain astrovirus strain into the newly formed bat colony after hibernation. Upon formation, the colony seemed to display a sufficient size and density of susceptible bat hosts to support viral infection dynamics [38].

Detection rates of astroviruses in bats varied greatly depending on the study designs and the tested species, but they seem to be generally higher than the detection rates found in rodents [42]. Lacroix et al. tested a total of 1876 bats from Cambodia and Lao PDR comprising four families of the *Yinpterochiroptera* and two families of the *Yangochiroptera* suborder, with the highest number of positives found in *Myotis* bats [15]. Intriguingly, a total of 47 *Myotis* bat individuals were tested with 20 (42.6%) of them giving a positive result [15]. Chu et al. observed an overall positive rate of 46% in their study that covered nine different species with the highest detection rates in relatively small sample sizes from *Miniopterus schreibersii* (three out of three animals tested positive) and *Myotis pilosus* often called *Myotis rickettii* (10 out of 12 animals tested positive) [11]. By using the same molecular approach at various sites in China, Zhu et al. observed an overall detection rate of 44.8% in 500 sampled bats [22]. This study revealed significant differences in detection rates between bat species, but very low differences between the analyzed sampling sites [22]. In contrast, Xiao et al. detected a lower percentage of positives among the 321 bats that were tested in their study, with the highest species-specific rate of 36.3% found in 55 *Miniopterus schreibersii* bats in a certain region of China [23]. Studies on European bats revealed relatively low overall detection rates. Nonetheless, high detection rates were observed in certain species such as *Miniopterus schreibersii* (up to 80%), *Myotis daubentonii* (up to 64%) or *Myotis nattereri* (up to 40%) [19,20,21].

With regards to the similarity level of the astrovirus sequences detected in bats, the influence of environmental factors, geographical vicinity and species affiliation has been discussed and is the subject of ongoing research. In all studies, there was a generally high degree of sequence variation among bat astroviruses [11,15,19,20,21,22,23,38,39,40]. Some of those astrovirus sequences showed only little host restriction and clustered with those detected in multiple bat families or other mammalian species; while other sequences seemed to be restricted to certain bat species and clustered into monophyletic groups [11,19,22,23]. Chu et al. reported to find mainly two subgroups of astroviruses in bats, which were subgroup A from *Miniopterus* species and subgroup B from *Myotis* species. Besides that, some bat-derived sequences clustered with human astroviruses, although with only weak statistical support [11]. While sampling bat colonies in China, Zhu et al. detected several virus strains within one roost in a single cave at the same sampling day [22]. However, some sequences from the same species collected at different locations were detected that clustered together in phylogenetic analyses [22]. Another study from Germany revealed similar astrovirus sequences detected in bats from the same species in different habitats more than 600 km apart that clustered within one group, indicating a host restriction irrespective of the location [19]. Besides these bat species-specific astrovirus strains reported in this study, there was still a high genetic diversity of astroviruses observed in bats of the same species at the same habitat at one time point, suggesting the circulation of multiple strains within the population, which is in accordance to other studies [11,15,22,23]. Bat astroviruses recently found in bats in Gabon showed to be genetically diverse with low host restriction, but distinct from astroviruses infecting other mammals [40]. A recent study investigating frugivorous and insectivorous bats in Lao PDR and Cambodia revealed a varying degree of host specificity in the detected astrovirus sequences. On the one hand, some sequences were only detected in individuals of one bat genus in phylogenetic analysis, while another sequence cluster seemed specific for insectivorous bats. In contrast, some sequences clustered together in a group that contained several sequences from different bat genera [15]. Interestingly, these authors found sequences with a close relationship to ungulate and porcupine hosts as well as two bat-derived sequences that shared 84%–87% of amino acid sequence identity with known murine astroviruses in the partial RdRp gene [15]. Similar findings had been reported from German and Chinese bat colonies where partial astrovirus sequences found in bats were more closely related to fox, murine, ovine, mink, human and even avian astroviruses than to other bat astroviruses in phylogenetic analyses [19,22,23]. Taken together, the current data indicate that bats may carry both, host restricted astroviruses as well as more diverse strains that may cluster together with other important members of the *Mamastrovirus* genus and even with members of the *Avastrovirus* genus. However, more detailed sequence data will be necessary for further analyses and to predict the potential for recombination or cross-species transmission.

## 3. Astroviruses Detected in Livestock

A number of recent reports have underlined the relevance of astrovirus infections also in livestock populations. Although summarizing all available data on astrovirus detection in mammalian livestock would by far exceed the scope of this review article, we here give a short overview on the current state of knowledge regarding bovine, ovine and porcine astroviruses (BoAstV, OvAstV, PoAstV). These species are highlighted because of the recent detection of novel BoAstV and OvAstV strains causing encephalitis [43,44,45,46,47,48], and the sequence similarities observed when comparing PoAstV strains with other mammalian strains including human astroviruses [49,50]. These animal species live in close contact to humans and might therefore play a role in the transmission of astroviruses that are not strictly species-specific.

Astrovirus-like particles in fecal samples from cattle were first reported in England in 1978 [50]. At that time, BoAstV were not associated with diarrhea or clinical symptoms in cattle, but rather linked with asymptomatic infections [51,52,53]. These early studies revealed a considerable antigenic diversity among BoAstV isolates. More than 25 years later, these findings were complemented by the description of the genetic diversity of BoAstV present in cattle in Hong Kong, with at least three different genotypes present in the population and co-infections by different genotypes in the same host at the same time [3]. Recently, another study from Japan revealed up to 15 different astrovirus-related sequences by using a metagenomics approach, but the presence of certain virus strains could not be correlated to a certain clinical picture in the analyzed animals [54]. Interestingly, the authors identified different phylogenetic clusters while comparing the different ORFs detected in their study with previously published BoAstV strains from the public database. Most of the novel strains clearly clustered in all analyzed ORFs with other BoAstV strains from China, forming the tentatively named lineage 1. A few strains clustered with an American BoAstV strain that was phylogenetically apart from these Asian sequences, and was thus tentatively named lineage 2. Notably, two other strains clustered within the lineage 1 when ORF1a and ORF1b were phylogenetically analyzed, but showed a closer relationship to the American BoAstV lineage 2 when ORF2 was investigated. Subsequently, a recombination event was discussed, but could not be substantially proven [54]. Furthermore, past interspecies transmission events were postulated, since the partial sequences of one newly discovered bovine strain exhibited the closest relation to porcine and ovine astrovirus sequences from the database in all calculated trees [54].

In addition to that, the perception of astrovirus tissue tropism and virulence in cattle has changed within the last few years, since several cases of bovine encephalitis have been associated with astrovirus infections [10,43,44,45,46,47]. Almost in parallel, two groups of researchers from the USA and from Switzerland independently detected a bovine astrovirus in cattle by the metagenomics approach that was associated with neurological symptoms and encephalitis in these animals [45,47]. Retrospectively, additional cases of bovine encephalitis of unknown etiology were screened by specific RT-PCR and in situ hybridization. These studies clearly presented evidence for the presence of astrovirus-related RNA in neurons and at the site of pathological changes, supporting the hypothesis that BoAstV infection may lead to encephalitis in cattle [43,44,45,47]. When both full-length sequences from the USA and Switzerland were compared, a strikingly close relationship of up to 92% of nucleotide identity between these two strains and a clear difference to other BoAstV strains were observed, leading to the proposal of a new species named BoAstV CH13/NeuroS1 within the *Mamastrovirus* genus [43]. Interestingly, these novel sequences phylogenetically clustered with rare astrovirus isolates from encephalitis cases in humans [18] and in minks displaying the so-called shaking mink syndrome [55]. A metagenomics analysis of brain material from a 15-month old cow from Germany that was presented with encephalitis of unknown etiology revealed another BoAstV that was named BoAstV BH89/14, which turned out to be closely related to a second BoAstV strain from Switzerland named BoAstV CH15 that was detected in one animal that was co-infected with both strains from Switzerland CH13 and CH15 [10,46]. Very recently, two complete very closely related astrovirus genome sequences were identified from brain tissues of two sheep from the same flock in the United Kingdom that had suffered from neurological signs within 9 months. This strain turned out to be closely related to the astrovirus strain, causing encephalitis in cattle [48]. Taken together, these findings provide strong evidence that certain strains of BoAstV and OvAstV may cause encephalitis in cattle and sheep. However, virus isolation is still lacking, which is essential to enable an experimental infection of animals and induce the disease for final proof of causation.

There is strong evidence that multiple astrovirus sero- and subtypes may infect pigs. However, the clinical significance of this finding is still doubtful, since various samples were collected from healthy pigs [13,56]. However, multiple studies from South Africa [57], Canada [13,49], USA [50,58], Colombia [59], China [60], Croatia [56,61], Czech Republic [62], Germany [63] and Hungary [12] have described several novel astroviruses to be circulating in the pig population. Astrovirus partial and full-length sequences were detected in fecal and extraintestinal samples such as serum [12,13,49,56,57,58,62]. There is a remarkably high fecal prevalence of astroviruses in pigs combined with a broad genetic diversity [13,56,59,61]. At least five lineages (PoAstV1-5) were identified to circulate in the investigated area of the USA with generally low sequence similarities [50]. Frequent co-infections of individual pigs with multiple astrovirus strains were found that might favor recombination events [50]. Phylogenetic links to astroviruses linked to other animal species were observed that could imply past cross-species transmission and recombination events, which might have included cattle, roe deer, mink, cats, humans and potentially other species [13,50,56,59,61]. Although the clinical significance for pigs remains to be investigated, these recent findings underline the importance of pigs as a porcine astrovirus reservoir.

## 4. Human Astroviruses (HAstV)

Human astroviruses (HAstV) have been classically divided into eight serotypes by immune electron microscopy and neutralization tests [4,64]. However, according to the latest classification scheme by the ICTV, recent characterizations of novel astrovirus isolates have been mainly based on the amino acid sequence of ORF2 that encodes the structural capsid polyprotein and is considered the most variable region of the astrovirus genome [4,65]. HAstVs are mainly known to cause diarrhea and gastrointestinal symptoms in young children as well as in elderly or immunocompromised patients [17]. Through the application of metagenomics analyses, novel astrovirus genotypes were revealed [8,9]. The first novel genotype was discovered in Melbourne, Australia, in a young patient with acute diarrhea. The virus named AstV-MLB1 was uncovered by metagenomic analysis and showed to be highly divergent from any of the previously known HAstVs [8]. Interestingly, a newly discovered rat astrovirus from China detected almost simultaneously seemed to form a sister clade to the HAstV-MLB1 [42]. At the same time, more HAstV genotypes were discovered during an outbreak of sporadic diarrhea in Virginia, leading to the formation of the novel AstV-VA1 genogroup [9]. Furthermore, Kapoor et al. identified astrovirus sequences from stool samples of patients from Nepal, Pakistan and Nigeria with symptoms of either gastroenteritis or non-poliovirus acute flaccid paralysis (AFP) [66]. The majority of the detected astrovirus sequences clustered with classic HAstV, whereas four sequences from Nigeria were most closely related with previously described AstV-MLB1 [66]. A total of eight novel astrovirus sequences were detected in patients with AFP or gastrointestinal symptoms as well as in healthy control patients, indicative of a multifactorial disease or an astroviral pathogenicity that is dependent on the patient’s health status. Intriguingly, these sequences showed a significant clustering with mink and ovine astroviruses, resulting in the abbreviation of HMOAstV for human, mink and ovine-like astrovirus [66]. However, the authors stated a close relationship to AstV-VA1. Taking all these findings into account, there are at least three genotypes of astroviruses in humans, namely the classic HAstVs, AstV-MLB1 and AstV-VA1. Very recently, some of the novel astroviruses that are phylogenetically apart from the classic HAstV have been identified as the causative agents for cases of neurotropic infections in humans [18,67,68]. In two cases of children with X-linked agammaglobulinemia in the USA and in France, an astrovirus (named VA1/HMO-C) was detected in the brain tissues of the affected boys [18,68]. These astroviruses showed to be genetically distinct from classic HAstV and clustered with mink astroviruses and other astrovirus strains that have been recently reported in human cases of encephalitis [18,68]. Although a direct link could not be proven, Quan et al. interestingly described the affected patient to live in proximity to a mink farm [18]. Another case of astrovirus infection was reported in an 18-month-old child in the United Kingdom with encephalopathy where high virus titers were found in brain tissue and cerebrospinal fluid [67]. Phylogenetic analyses revealed a close relationship to previously described VA1/HMO-C astroviruses, further supporting the neurotropism of certain astrovirus strains in immunocompromised patients [67]. These three case reports prove the very wide geographic distribution of these neurotropic HAstV strains.

The co-circulation of three genotypes of astroviruses in humans might permit recombination events between two parent strains in the case of a co-infection. In the past, recombination events have been frequently reported to occur in human astroviruses. This mainly occurred in the genomic region between ORF1b and ORF2 [9,17,69,70]. As mentioned earlier, pigs were also shown to harbor diverse astroviruses enabling for recombination events. In a study where fecal samples from pigs and humans from several Colombian regions were collected, the authors assumed that recombination between the porcine and the human astroviruses within the variable region of ORF2 had occurred [59]. Notably, the authors concluded an anthropozoonotic viral transmission from humans to pigs due to the highly increased genetic diversity of human astrovirus strains compared to the porcine strains [59]. In addition, a complete porcine astrovirus genome that clustered within the PAstV3 lineage revealed 50.5%–55% of sequence identity with mink and HMO astrovirus strains but only 38%–42% with representatives of other PAstV lineages, which led the authors to speculate about zoonotic potential [50]. Interestingly, there is recent evidence suggesting that non-human primates (NHP) can harbor a wide variety of mammalian astrovirus genotypes with some of them previously only known to be associated with human infection [14]. Importantly, astrovirus sequences were detected in NHP that seemed to result from a recombination between human and animal genotypes [14]. Another study suggested an antigenic similarity between HAstVs and feline astroviruses, since a human serum reacted against a feline astrovirus in immune electron microscopy [71]. On the one hand, astrovirus sequences that were shown to be phylogenetically related to astrovirus strains associated with human infections have only been detected in very rare events in animals [14,43,47,55,59]. On the other hand, these findings suggest that recombination events between different mammalian parent strains have occurred and that the species barrier must have been crossed.

## 5. Phylogenetic Analyses and Future Directions

Recently, a broadly reactive semi-nested RT-PCR has been established to detect astrovirus-related RNA in bat samples. This PCR assay was used in different studies worldwide and targets the relatively conserved region of the RdRp gene [11]. However, to better evaluate the occurrence of different genotypes in bats, more sequence information will be necessary. According to taxonomic classifications, sequences of the capsid region encoded by ORF2 might be beneficial. Considering the high degree of sequence variation between the present astrovirus partial RdRp sequences, it might be challenging to develop specific molecular assays. One opportunity might be the N-terminal region of the ORF 2 that is considered to display a higher degree of conservation among the *Astroviridae* than the C-terminal region of the capsid protein, which seems to be highly divergent and responsible for serological strain specificity [72]. Therefore, the N-terminal region might serve as a valuable target region for PCR. In the past, Zhu et al. used ORF2 gene-specific primers for an extended sequencing of astroviruses in bats [22]. These gene sequences were phylogenetically analyzed and compared with the analyses of the RdRp genes. Results indicated that both bat-specific clusters as well as mixed clusters containing sequences from bats, mink and sheep may occur, which suggests potential interspecies transmission [22].

To date, no bat astrovirus isolate cultivated or propagated in cell culture has been reported. The chances of being successful are made narrower by multiple factors, such as appropriate cell lines and suitable sample quality and quantity, which are needed for successful virus isolation. With regards to HAstV, it has been described that certain subtypes were successfully cultivated in cell lines that were refractory to other HAstV subtypes, indicating individual requirements for propagation [73]. Moreover, only very little is known about the astrovirus-specific cell attachment and entry [17]. Therefore, due to the great diversity of astroviruses assumed to be present in bats, it is not surprising that bat astroviruses have not been isolated yet. Full-length sequences of bat astroviruses which could provide more phylogenetic information are also not yet available. Only three-quarters of the genome of one bat astrovirus strain from China has been made available in the public database [11], thus hampering any further classification and analysis as it was performed for bovine, ovine, porcine and human astroviruses where several full-length genomes could be obtained [3,5,8,9,13,43,48,49,50,54,56,66,67].

Novel astroviruses have been identified by metagenomics approaches whenever diseases of unknown etiology have occurred e.g., in humans, cattle or sheep [18,45,47,48]. Metagenomics have been widely used as an unbiased system to obtain potentially causative viral sequences in case standard procedures such as PCR fail to reveal the causative agent. Furthermore, it can be utilized to obtain more sequence information or even full-length sequences. With regards to the lack of bat astrovirus isolates, metagenomics might be a suitable alternative to gain more detailed sequence information for further analyses. Nonetheless, small sample sizes and low virus titers in the samples might also impede these approaches.

## 6. Zoonotic Potential and Risk Assessment

Besides the classical course of astrovirus infections resulting in diarrhea, there have been several recent reports of astrovirus infections causing encephalitis cases in humans, cattle and sheep [10,18,43,44,46,48,67,68]. A study investigating fecal samples from NHP for the presence of astrovirus-related RNA revealed that NHP carried astroviruses showing up to 100% sequence identity within the RdRp gene to HAstV-1 [14]. In addition to that, serological assays confirmed the presence of specific antibodies against HAstV-1 and MLB in these animals [14]. These findings somehow challenge the traditional paradigm of the species-specificity of astroviruses. Bats have been found to carry a broad variety of viruses with some of them being considered zoonotic agents. Astroviruses in bats have been investigated and phylogenetically characterized since 2008, resulting in the detection of a wide geographic distribution, a great genetic diversity and a varying degree of host restriction.

The ICTV defined an amino acid sequence diversity in the capsid gene of <0.312 and >0.378 within and between astrovirus species, respectively. These rules cannot be applied in sensu stricto towards bat-derived astrovirus sequences since most of the bat astrovirus sequences originated from the semi-nested RT-PCR established by Chu et al. [11] that targets the highly conserved RdRp gene for which there is no range of sequence diversity determined by ICTV. Moreover, most of the bat astroviruses show a great diversity that would technically qualify them as different species, but they still cluster together in monophyletic clades. Another problem that was recently raised by Karlsson et al. is that some bat-derived astrovirus sequences were published and used for phylogenetic analyses in certain studies but not made available to the research community in the public database which might bias the results of future analyses [14,22]. Expanded molecular assays, full-length genomes or virus isolates would greatly improve the situation.

With regards to the zoonotic potential of bat-derived astroviruses, it needs to be highlighted that neither any successful attempt of virus isolation nor any full-length sequences from bat-associated astroviruses have been published so far. While analyzing a PCR product from the highly conserved region within the RdRp, the genetic diversity of bat astroviruses seems to be remarkable. Therefore, virus isolation attempts and/or metagenomics approaches are crucial to further investigate and classify the bat astrovirus diversity. However, many virus isolation attempts for bat astroviruses failed in the past which might be explained by very inefficient virus shedding or by insufficient sample quality.

The co-occurrence of different strains within bat colonies might favor the potential of co-infection of individuals with multiple strains at the same time. Therefore, recombination events between different bat strains seem possible. Virus recombination events may lead to novel virus strains to which the affected host may have a lower immunity than to one of the parent strains. At some point, it might even enable these novel viruses to potentially cross species barriers. There has been a close genetic relationship observed between certain mammalian astrovirus sequences derived from humans and rats as well as between human, ovine and mink astroviruses [42,66]. Evidence for recombination events in other mammals such as humans, NHP, cattle and pigs has already been described [14,54,59,70]. Although astroviruses have been considered relatively species-specific in the past, the incidence of recombination events combined with widespread infections across many animal species and the high genetic diversity may generally permit the emergence of novel astroviruses from certain hosts with zoonotic potential. Concerning bats, to date, there has been no case ever reported of zoonotic astrovirus transmission.

## 7. Conclusions

To assess and predict the zoonotic potential of bat astroviruses, we need a better understanding of the astrovirus infection dynamics, including the shedding and transmission of viruses between bats and potentially from bats to other mammalian species. Therefore, it will be important to conduct more extensive surveillance studies of different bat species in different habitats as well as consecutive studies on well-characterized colonies followed over long periods of time. For optimal results and the greatest benefit, an interdisciplinary approach would be desirable where virologists, bat ecologists and zoologists work closely together. Novel sequencing techniques might deliver more detailed data on phylogenetic relationships of astroviruses from bats and other hosts, but they should always be combined with field data for the best possible interpretation of their significance. There is strong evidence that astroviruses in general are not as species-specific as they have been considered to be in the past. However, sequence data from bat astroviruses are still insufficient to really assess zoonotic potential. To overcome this issue, virus isolates and more detailed sequence information from several strains that are thought to circulate in bat populations would be most helpful. Although bats are frequently proposed as the natural reservoir of some highly pathogenic and zoonotic viruses, the overall risk for astrovirus transmission from bats to humans is assumed to be relatively low and does not, to our current understanding, exceed the risk of an astrovirus transmission from other mammals such as minks, pigs or cattle to humans.

## Figures and Tables

**Table 1:** Species and number of bats that were investigated for the presence of astrovirus-related RNA/genome by semi-nested reverse transcription PCR (RT-PCR).

Family	Species	Region	Tested Animals	Positive Results (%)	Reference
Vespertilionidae	*Barbastella barbastellus*	Hungary	13	0 (0%)	[20]
	*Eptesicus nilssonii*	Czech Republic	1	0 (0%)	[39]
	*Eptesicus serotinus*	Hungary	3	0 (0%)	[21]
		Hungary	7	0 (0%)	[20]
		Czech Republic	1	1 (100%)	[39]
	*Hesperoptenus* sp.	Cambodia	1	0 (0%)	[15]
	*Hypsugo savii*	Czech Republic	4	1 (25%)	[39]
	*Ia io*	China	11	4 (36.4%)	[22]
		Lao PDR	32	1 (3.1%)	[15]
	*Miniopterus inflatus*	Gabon	155	16 (10.3%)	[40]
	*Miniopterus magnater*	China (Hong Kong)	122	67 (54.9%)	[11]
	*Miniopterus pusillus*	China (Hong Kong)	73	31 (42.5%)	[11]
	*Miniopterus schreibersii*	China (Hong Kong)	3	3 (100%)	[11]
		China	19	12 (63.2%)	[22]
		China	187	22 (11.8%)	[23]
		Hungary	15	12 (80%)	[20]
	*Myotis alcathoe*	Hungary	16	0 (0%)	[20]
	*Myotis bechsteinii*	Hungary	22	1 (4.5%)	[21]
		Hungary	125	5 (4%)	[20]
		Czech Republic	1	0 (0%)	[39]
		Germany	321	35 (10.9%)	[19]
	*Myotis brandtii*	Hungary	3	0 (0%)	[20]
	*Myotis blythii*	Hungary	2	0 (0%)	[21]
		Hungary	10	0 (0%)	[20]
	*Myotis chinensis*	China (Hong Kong)	9	3 (33.3%)	[11]
	*Myotis dasycneme*	Hungary	11	0 (0%)	[20]
	*Myotis daubentonii*	Hungary	7	3 (42.9%)	[21]
		Hungary	81	6 (7.4%)	[20]
		Czech Republic	3	0 (0%)	[39]
		Germany	47	30 (63.8%)	[19]
	*Myotis dasycheme*	Hungary	4	0 (0%)	[21]
	*Myotis emarginatus*	Hungary	5	1 (20%)	[20]
		Czech Republic	1	1 (100%)	[39]
	*Myotis horsfieldii*	Cambodia	47	20 (42.6%)	[15]
	*Myotis myotis*	Hungary	6	0 (0%)	[21]
		Hungary	29	0 (0%)	[20]
	*Myotis mystacinus*	Hungary	1	0 (0%)	[20]
		Czech Republic	1	1 (100%)	[39]
	*Myotis nattereri*	Hungary	4	0 (0%)	[21]
		Hungary	37	1 (2.7%)	[20]
		Germany	248	99 (39.9%)	[19]
	*Myotis pilosus*	China (Hong Kong)	12	10 (83.3%)	[11]
		China	16	2 (12.5%)	[22]
		China	1	0 (0%)	[23]
	*Myotis* spp.	China	5	3 (60%)	[22]
	*Nyctalus leisleri*	Hungary	6	0 (0%)	[20]
	*Nyctalus noctula*	Hungary	14	4 (28.6%)	[20]
		Czech Republic	7	1 (14.3%)	[39]
	*Nyctalus plancyi velutinus*	China	1	0 (0%)	[22]
	*Pipistrellus abramus*	China (Hong Kong)	2	1 (50%)	[11]
		China	20	1 (5%)	[22]
	*Pipistrellus nathusii*	Hungary	3	0 (0%)	[20]
		Czech Republic	1	0 (0%)	[39]
		Germany	22	6 (27.3%)	[19]
	*Pipistrellus pipistrellus*	Hungary	1	0 (0%)	[21]
		Hungary	12	0 (0%)	[20]
		Czech Republic	12	1 (8.3%)	[39]
		Germany	7	0 (0%)	[19]
	*Pipistrellus pygmaeus*	Hungary	6	1 (16.7%)	[20]
		Czech Republic	1	1 (100%)	[39]
		Germany	12	6 (50%)	[19]
	*Pipistrellus* spp.	China	5	0 (0%)	[22]
		Cambodia	29	0 (0%)	[15]
	*Plecotus auritus*	Hungary	11	1 (9.1%)	[21]
		Hungary	29	1 (3.4%)	[20]
		Czech Republic	2	0 (0%)	[39]
		Germany	118	24 (20.3%)	[19]
	*Plecotus austriacus*	Hungary	3	0 (0%)	[20]
		Czech Republic	2	0 (0%)	[39]
	*Scotophilus kuhlii*	China	38	6 (15.8%)	[23]
		China	2	0 (0%)	[22]
	*Scotophilus* spp.	Cambodia	524	39 (7.4%)	[15]
	*Tylonycteris pachypus*	China	2	0 (0%)	[22]
	*Tylonycteris* sp.	Cambodia	1	0 (0%)	[15]
	*Vespertilio murinus*	Hungary	3	0 (0%)	[20]
		Czech Republic	5	1 (20%)	[39]
	**TOTAL**		**2468**	**469 (19%)**	
Rhinolophidae	*Rhinolophus affinis*	China	2	0 (0%)	[22]
	*Rhinolophus euryale*	Hungary	3	0 (0%)	[20]
	*Rhinolophus ferrumequinum*	China	7	0 (0%)	[23]
		China	4	2 (50%)	[22]
		Hungary	12	0 (0%)	[20]
	*Rhinolophus hipposideros*	Hungary	3	0 (0%)	[20]
		Czech Republic	2	1 (50%)	[39]
	*Rhinolophus lepidus*	China	11	0 (0%)	[23]
	*Rhinolophus macrotis*	China	2	0 (0%)	[23]
		China	1	0 (0%)	[22]
	*Rhinolophus pearsonii*	China	1	1 (100%)	[22]
	*Rhinolophus rouxii*	China (Hong Kong)	8	1 (12.5%)	[11]
	*Rhinolophus sinicus*	China	1	0 (0%)	[22]
	*Rhinolophus* sp.	Cambodia	53	1 (1.9%)	[15]
		Lao PDR	102	4 (3.9%)	[15]
	**TOTAL**		**212**	**10 (4.7%)**	
Hipposideridae	*Aselliscus stoliczkanus*	China	1	0 (0%)	[22]
	*Aselliscus* sp.	Lao PDR	7	0 (0%)	[15]
	*Hipposideros armiger*	China (Hong Kong)	10	0 (0%	[11]
		China	109	21 (19.3%)	[22]
	*Hipposideros gigas*	Gabon	226	7 (3.1%)	[40]
	*Hipposideros larvatus*	China	29	4 (13.8%)	[22]
		China	1	0 (0%)	[23]
	*Hipposideros pomona*	China	95	13 (13.7%)	[22]
		China	15	0 (0%)	[23]
	*Hipposideros ruber*	Gabon	394	17 (4.3%)	[40]
	*Hipposideros* spp.	Cambodia	4	1 (25%)	[15]
		Lao PDR	26	1 (3.8%)	[15]
	**TOTAL**		**917**	**64 (7.0%)**	
Pteropodidae	*Cynopterus sphinx*	China (Hong Kong)	11	0 (0%)	[11]
	*Cynopterus* spp.	Cambodia	321	0 (0%)	[15]
		Lao PDR	19	0 (0%)	[15]
	*Eonycteris* sp.	Cambodia	28	0 (0%)	[15]
		Lao PDR	51	3 (5.9%)	[15]
	*Macroglossus* sp.	Cambodia	21	0 (0%)	[15]
		Lao PDR	1	0 (0%)	[15]
	*Megaerops* sp.	Cambodia	29	0 (0%)	[15]
		Lao PDR	69	0 (0%)	[15]
	*Pteropus* sp.	Cambodia	10	0 (0%)	[15]
	*Rousettus aegyptiacus*	Gabon	162	2 (1.2%)	[40]
	*Rousettus leschenaultia*	China	59	1 (1.7%)	[23]
	*Rousettus* sp.	Cambodia	11	1 (9.1%)	[15]
		Lao PDR	322	23 (7.1%)	[15]
	**TOTAL**		**1114**	**30 (2.7%)**	
Emballonuridae	*Coleura afra*	Gabon	25	2 (8%)	[40]
	*Taphozous melanopogon*	China	172	160 (93%)	[22]
	*Taphozous* spp.	Cambodia	147	4 (2.7%)	[15]
	**TOTAL**		**344**	**166 (48.3%)**	
Megadermatidae	*Megaderma lyra*	China	1	1 (100%)	[22]
		Cambodia	21	2 (9.5%)	[15]
	**TOTAL**		**22**	**3 (13.6%)**	
